# Dynamic U-shaped convolutional network for mouse cardiac image segmentation and quantification

**DOI:** 10.3389/fmed.2026.1836537

**Published:** 2026-05-28

**Authors:** Yu Wang, Wenwen Zhang, Wanjun Zhang, Cenbin Huang, Ming Zhang, Naian Xiao, Shengge Xu

**Affiliations:** 1 School of Physical Education, Henan University, Kaifeng, Henan, China; 2 Henan Key Laboratory of Big Data Analysis and Processing, School of Computer and Information Engineering, Henan University, Kaifeng, Henan, China; 3 Department of Pediatrics, Zhongshan Hospital Xiamen University, Xiamen, Fujian, China; 4 Department of Neurology, The Third Hospital of Xiamen, Xiamen, Fujian, China; 5 School of Nuclear Science and Engineering, Hefei University of Technology, Hefei, Anhui, China

**Keywords:** cardiac segmentation, cardiac slice image, mouse cardiac, myocardial infarction, U-shape convolutional

## Abstract

Accurately segmenting and quantifying mouse cardiac slice images with myocardial infarction is of great significance in cardiovascular disease research. Manual methods are time-consuming, so automatic segmentation is highly sought after. However, due to the irregular U-shaped structure of infarcted and high-risk areas, the task is complex. This study proposes a dynamic U-shaped convolutional network that adapts to these irregular structures. We designed a dynamic convolution to focus on U-shaped local features, and developed a dual-stream fusion block to enhance performance. An attention gate mechanism suppresses irrelevant information, highlighting key features. We constructed a dataset of 243 mouse cardiac slice images with myocardial infarction. The proposed method outperforms other models, achieving an average Dice coefficient of 80.68%, 1.7% higher than the best existing algorithm. For infarct size segmentation, it reached 80.13%, surpassing the current optimal method by 2.43%. Additionally, our approach can quantify the ratio of infarcted to risk areas, aiding the assessment of myocardial injury severity. The dataset is available at https://github.com/wwz4416/mouse-cardiac-dataset.

## 1 Introduction

**Figure 1:**
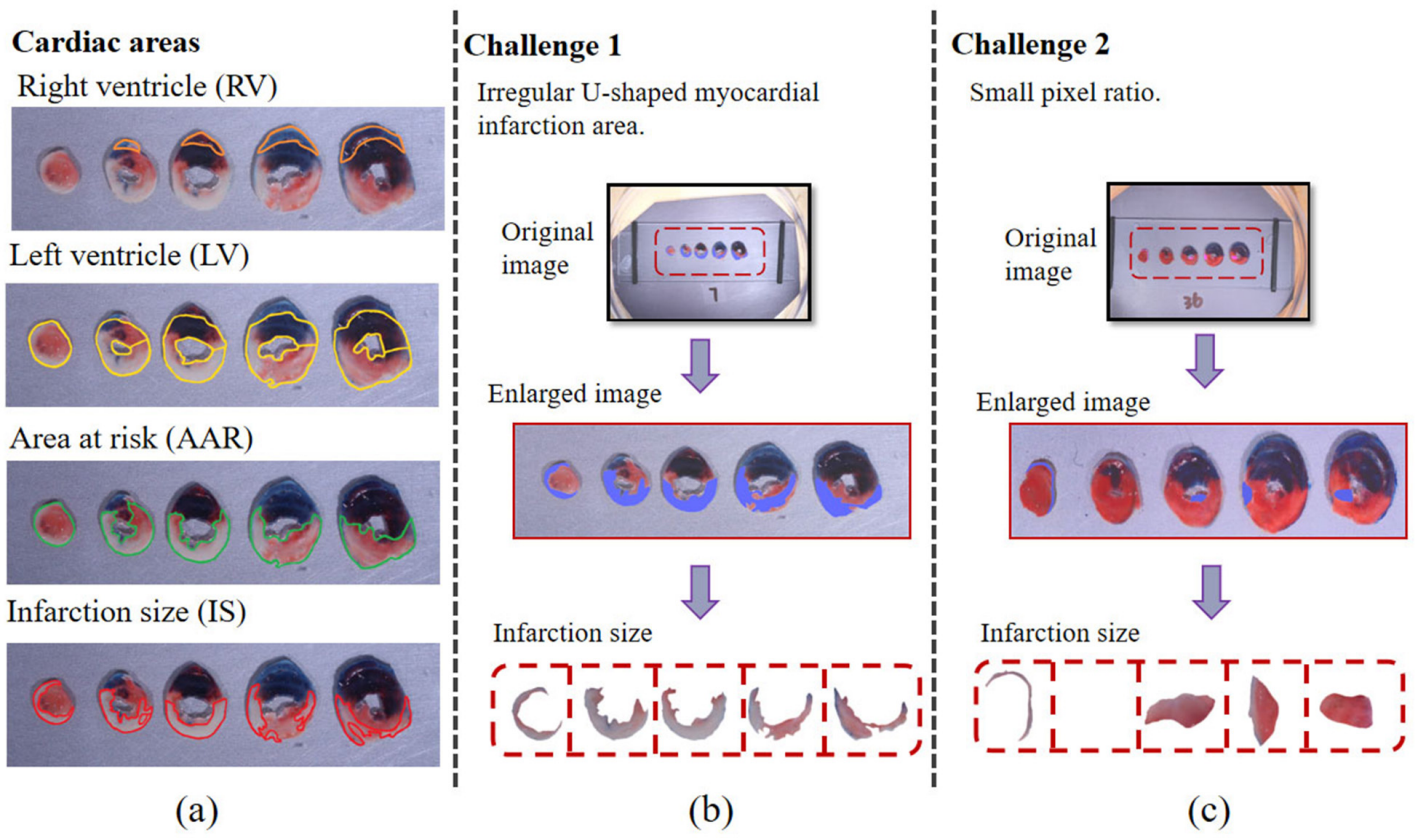
**(a)** Mouse cardiac illustration. Including Right ventricle (RV), left ventricle (LV), Area at risk (ARR), and infarct size (IS). **(b) Challenge 1**. The infarcted area presents an irregular U-shape. **Motivation:** Design convolution kernels that adapt to U-shaped targets. **(c) Challenge 2**. The proportion of infarcted areas is small and may not exist. **Motivation:** Designed a model to achieve precise segmentation. To clearly display the structure of different regions, we enlarged the mouse image.

To fully understand the pathogenesis of myocardial infarction and age-related diseases, mouse cardiac modeling performed as a critical step (1–3). To ensure the accuracy of the experiment, multiple sets of experiments are required to analyze the areas of the right ventricle (RV), left ventricle (LV), area at risk (AAR), and infarct size (IS) (as is shown in Figure 1a) in mouse heart sections. Existing research mainly relied on manual operation by professionals on imaging software to measure LV, ARR and IS, resulting in slow processing speed and susceptibility to subjective factors (4). Therefore, designing an automated segmentation and quantification model for mouse cardiac slice images is of great significance and necessity. However, the existing cardiac segmentation models mainly focus on the study of the human cardiac (5, 6), while there are few studies related to the automatic intelligence-assisted segmentation and quantification of mouse cardiac slice images.

However, mouse cardiac slice images measurement is challenging, due to the following difficulties: (1) The IS is usually complex and irregularly U-shaped, as shown in Figure 1b. Traditional convolution typically assumes that the target region has a simple shape or boundary (7). However, the complex shapes of IS often exceed the processing capabilities of traditional segmentation techniques. Therefore, effectively capturing the features of irregular U-shaped infarcted areas has become a key challenge in current model design. (2) The target area is small and Control group slices may not contain the infarcted portion. Due to the small size of the mouse and its cardiac slices, the background occupies a larger area in the slide image, while the cardiac slices appear smaller. Especially in the infarcted area, it is not only limited in size but may also not exist in certain slices, as shown in Figure 1c. (3) In clinical applications, processing mouse cardiac slices often relies on manual analysis, which is not only time-consuming and tedious but also prone to high subjectivity, resulting in poor reproducibility (8). In the face of this situation, it is needed to design a robust model to accurately identify and segment the target area. This further emphasizes the need for automated quantification of infarct area.

Recently, to address the morphological challenges in image segmentation, many research teams have proposed various models to enhance the understanding of specific structures. Considering the shape features of tree crowns (9), tree instances are segmented efficiently by capturing the geometric shape features of the crown contour. Cross-view Aligned Segmentation Network (10) graph-based network successfully segments individual multiview patches along the surface of the knee joints with the help of the knee joint map. Patch segmentation is pieced together into a complete knee segmentation. Dynamic Snake Convolution Network (DSCNet) (11) presents a dynamic snake convolution that accurately captures the features of tubular structures, adaptively focusing on elongated and convoluted local structures. Zhang et al. (12) present a new morphology-inspired approach for segmenting gland images. These findings show that significant progress has been made in the field of image segmentation using morphological knowledge.

In this study, we propose a Dynamic U-shaped convolution Network (DUCNet) dedicated to image segmentation of mouse cardiac slices. Firstly, to focus on the irregular U-shaped local structures in the IS, we propose Dynamic U-shaped Convolution (DUConv) to enhance the perception of morphological features. Compared to Dynamic Snake Convolution (DSConv) (11), our proposed DUConv considers the irregular boundaries that IS presented and is able to adaptively learn and pay attention to the U-shaped features, thus improving the segmentation performance of IS. Secondly, we design Dual-Stream fusion (DSF) block to address the challenges of small pixel percentages in the target area and possible missing IS in the image. This module takes advantage of DUConv and aims to perceive the U-shaped structural features. It enhancing the model's ability to perceive the target regions and improve the extraction efficiency of target features. Meanwhile, we introduce the residual structure to learn the details and edge features of the target region to further enhance the recognition and segmentation performance.

The contributions of our work are summarized as follows :

(1) We constructed a dataset of cardiac slices from mice with myocardial infarction and expert annotated three regions with fine details: left ventricle, AAR and IS. To our knowledge, this dataset is the first of its kind. The dataset will be available via GitHub.
(2) We proposed a dynamic U-shaped convolution that adaptively focused on irregular U-shaped local features. Based on this, we designed a dual-stream fusion module aimed at effectively addressing the precise segmentation of small regions. Experimental results demonstrated that the proposed method achieved an average Dice coefficient of 80.68% on the mouse cardiac tissue dataset.
(3) The contribution of the application. Traditional manual analysis of mouse cardiac slices often involves strong subjectivity. The DUCNet algorithm can robustly achieve automated quantitative analysis of infarct size.

This paper is organized as follows. Section 2 reviews related work. Section 3 describes the proposed network. Section 4 presents the experimental results. Finally, section 5 gives the conclusion and discussion of the paper.

## 2 Related works

In this section, we reviewed previous relevant studies. Firstly, we reviewed the medical image segmentation algorithms (Section 2.1). Next, we discussed the design of convolutional (Section 2.2) in detail to understand the importance of different requirements required for the design of different convolutions.

### 2.1 Medical image segmentation

Cardiac image segmentation is essential for accurately extracting structural information of the heart, aiding in precise diagnosis and personalized treatment planning (13). The application of deep learning technology in the field of medical imaging has attracted widespread attention, and automatic recognition and segmentation of lesions in medical images have become one of the core issues of concern for many researchers.

To address this challenge, Ronneberger et al. (14) proposed U-Net at the MICCAI Conference 2015. This structure is a U-shaped network with skip connections, which is widely used due to its excellent performance in medical image segmentation. ResUNet (15) extends the U-Net architecture by introducing residual connections, enabling the network to train effectively even with a large number of layers, thereby improving its ability to capture complex features of medical images. In addition, Oktay et al. (16) proposed a novel attention gate (AG) model that can indirectly learn to suppress irrelevant regions in medical images and highlight salient features suitable for specific tasks through training, thus overcoming the limitations of using external tissue or organ localization units in traditional CNNs (17). In order to further improve segmentation accuracy, UNet++ (18) was proposed. This model effectively reduces the semantic gap between the feature maps of the encoding and decoding subnetworks through a series of nested dense skip path connections. UNET 3+ (19) was designed for organs of different sizes, utilizing full-size skip connections and deep supervision, combined with details and semantic information from different feature scales, to learn hierarchical representations from full-size aggregated feature maps. As a powerful adaptive framework, nnU-Net (20) considers the specific properties of computer hardware functionality and datasets in its self-configuring mechanism for automatic segmentation framework. VM-Unet (21) introduces Visual State Space as the foundational block for capturing broad contextual information. Recently, EEMSNet (22) has shown excellent performance in cardiac segmentation tasks, utilizing the edge supervision module on the encoder to improve segmentation performance significantly.

### 2.2 Convolution strategy

In the research of CNNs, the design of convolutional kernels is a crucial task. Traditional convolutional kernels typically have fixed sizes and sizes. Based on this design, traditional CNNs gradually reduce the resolution of the image until only weak spatial information feature maps are retained. However, Dilated Convolutions (23) have emerged with the increasing demand for larger receptive fields and richer contextual information. This convolution operation can maintain the original receptive field of the network without losing the spatial resolution of the image. Dai et al. (7) proposed Deformable Convolution, which can adjust its shape according to actual situations and better extract input features. But this kind of convolution is difficult to focus on specific structures. To adapt to slender and complex tubular structures, DSCNet proposed a DSConv (11). This convolution introduces deformation offset. In addition, to ensure that the perception field does not deviate from the target, DSCNet adopts an iterative strategy.

In summary, although the existing segmentation networks and the exploration of convolutions have achieved nontrivial progress, they cannot be directly applied to mouse slice segmentation tasks. Based on the exploration and understanding of convolution operations mentioned above, we propose a novel U-shaped dynamic convolution. This convolution is designed based on the irregular U-shaped local structural features of the target area and the flexibility of dynamic convolution, which can effectively enhance the perception of U-shaped features.

## 3 Method

In this section, we first present the overall architecture of the proposed DUCNet. In Section 3.2, we provide a detailed introduction to DUConv. In Section 3.3, we describe the DSF blocks that constitute the encoder and decoder.

### 3.1 The overall architecture of DUCNet

**Figure 2:**
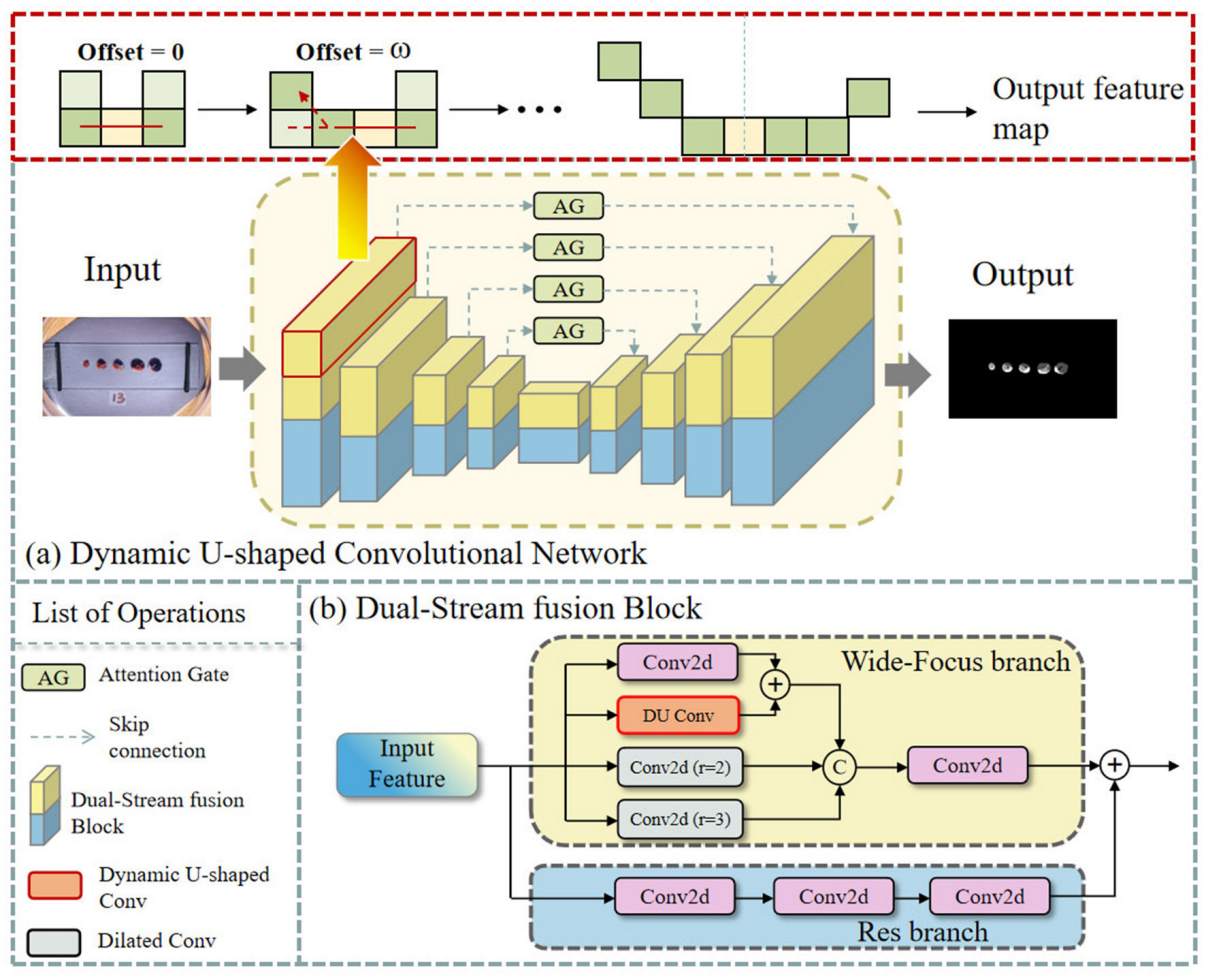
The proposed method is illustrated in the segmentation diagram of mouse cardiac slice images. **(a)** Dynamic U-shaped convolution (DUConv) adaptively focuses on irregular U-shaped local features through deformation offset. **(b)** Architecture of the proposed DUCNet. **(c)** Structural diagram of dual stream fusion module.

Figure 2 shows the overall architecture of DUCNet. Specifically, as shown in Figure 2a, DUCNet includes encoder, decoder and skip connections. We adopted a symmetric structure similar to U-Net (14). The basic unit of DUCNet is the DSF block.

The original image is mapped to C channels through convolutional layers. The feature map generated by this process is $x\in R^{H\times W\times C}$. Next, it was fed into the encoder for feature extraction. The encoder consists of four stages, each containing a DSF block. The number of channels in each stage gradually increases, in order of [C, 2C, 4C, 8C]. Apply maximum pooling at the end of each stage to reduce the height and width of input features.

The decoder takes the bottleneck representation as its input. The decoder is also organized into four stages, each containing a DSF block, with channel counts of [8C, 4C, 2C, C] for each stage. At the end of each stage, upsampling operations are used to increase the height and width of input features.

In terms of skip connections, we used Attention Gate (AG) (16). The feature map of the decoder and the feature map of the encoder in the previous layer are used as inputs for AG. Splice the AG processed results with the upsampled decoder section. This block can indirectly learn to suppress irrelevant regions in mouse cardiac slice images through training, and highlight significant features of segmentation tasks.

### 3.2 Dynamic U-shaped convolution

In order to improve the flexibility of convolutional kernels in capturing complex geometric features of targets, we were inspired by deformable convolutions (7) and introduced a deformable offset. This enables convolutional kernels to weigh input features at different positions, thereby more effectively capturing the shape and structural information of the target. However, if the model learns these offsets completely freely, the perception range often deviates from the target, especially when dealing with U-shaped structural features. Inspired by DSCNet (11), in order to further improve the model's focusing ability on the target area, we adopted an iterative strategy to optimize the offset. Specifically, to ensure that the receptive field can effectively present a U-shaped structure, we set the initial offset of the center point and the neighbor points around it to zero. Starting from the second grid point, the model will gradually optimize these offsets based on the current offset and feedback from the target area. In addition, to maintain symmetry, we also ensure that the offset of grid points symmetrical to the center point maintains the same sign as much as possible, that is, both positive and negative values. The change in the *x*-axis direction is shown in Equation 1. The change in the *y*-axis direction is shown in Equation 2.


$$\begin{array}{l} G_{i\pm c}^{x}=\left(x_{i}+c,y_{i}+\omega \overset{i+c}{\underset{i}{\sum }}\Delta y_{i}\right),c\in \left[-3,3\right] \end{array}$$ (1)

The change in the *y*-axis direction is:


$$\begin{array}{l} G_{j\pm c}^{y}=\left(x_{j}+\omega \overset{j+c}{\underset{j}{\sum }}\Delta x_{i},y_{j}+c\right),c\in \left[-3,3\right] \end{array}$$ (2)

where ω represents weight. When *c* = 1, the value of ω is 0; When *c* takes other values, the value of ω is 1. This process optimizes the observation position of each target area and determines the position of the next grid. By adjusting this strategy, the offset gradually increases from the center value toward the boundary value, forming a gradually widening U-shaped structure. This U-shaped structure not only ensures continuous attention to the target area but also effectively avoids excessive expansion of the perception range. The position of a pixel is represented by coordinates (*x*_*i*±*c*_, *y*_*j*±*c*_), where *c* represents the horizontal distance relative to the central lattice, and the value range of *c* ∈ [0, 1, 2, 3]. The selection of lattice positions in convolutional kernels is a gradual accumulation process. Starting from the second grid at $G_{i+2}^{x}$ or $G_{i-2}^{x}$ and $G_{j+2}^{y}$ or $G_{j-2}^{y}$, each grid increases its position relative to the previous grid by an offset ▵. The value range of ▵ being {δ|δ∈[−1, 1]}.

**Figure 3:**
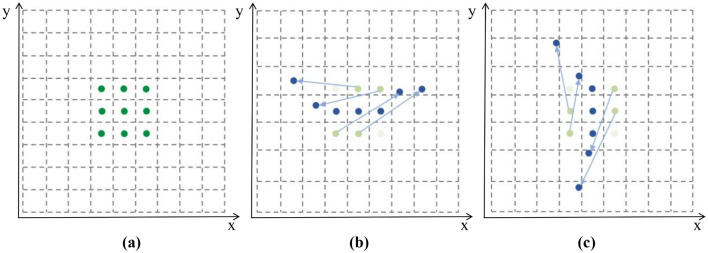
The sampling methods of normal convolution and dynamic U-shaped convolution with a kernel size of 3 × 3 were demonstrated. **(a)** The figure shows nine points (green dots) sampled according to the normal convolution pattern. **(b)** The point shown is the sampled dynamic U-shaped convolution in the *x*-axis direction (blue). **(c)** The point shown is the dynamic U-shaped convolution sampled in the *y*-axis direction (blue).

In DUConv, we use a convolutional kernel of size 7. As shown in Figure 3, DUConv selects sampling points based on the standard convolutional kernel. It is worth noting that during the linearization process of the standard convolutional kernel, we discard the two most distant sampling points, removing the farthest two points from the center along the *x*-axis and *y*-axis directions. This not only reduces redundant information, helping the convolutional kernel focus better on the key areas of the target, but also effectively reduces the computational cost.

### 3.3 Dual-stream fusion block

The DSF block is constructed based on a Wide-Fouse (WF) branch and a residual (Res) branch. As shown in Figure 2b, the WF branch contains DUConv and two types of dilated convolutions with dilation rates of 2 and 3, respectively. DUConv is mainly responsible for extracting local U-shaped features, while dilated convolution captures a wider range of feature information by expanding the receptive field. The feature maps of these three convolutions are concatenated. Subsequently, the concatenated features are generated through feature fusion. The Equation 3 can represent this process.


$$\begin{array}{l} WF\left(\hat{x}\right)=\text{Conv}\left(\text{Concat}\left(\text{DU}\left(\hat{x}\right),{\text{DL}}_{2}\left(\hat{x}\right),{\text{DL}}_{3}\left(\hat{x}\right)\right)\right) \end{array}$$ (3)

where $\hat{x}$ represents the input feature. The output feature after DU convolution is $DU\left(\hat{x}\right)$. And ${\text{DL}}_{2}\left(\hat{x}\right)$ and ${\text{DL}}_{3}\left(\hat{x}\right)$ represents the features after dilated convolution with dilation rates of 2 and 3, respectively.

The Res branch consists of three convolutional layers. In this branch, the input features are added to the output features of WF after passing through three convolutional layers. Ultimately, these features are reformed and propagated to the next layer. This process is implemented through the Equation 4.


$$\begin{array}{l} DSF\left(\hat{x}\right)={\text{Conv}}_{3}\left({\text{Conv}}_{2}\left({\text{Conv}}_{1}\left(\hat{x}\right)\right)\right)+WF\left(\hat{x}\right) \end{array}$$ (4)

where $\hat{x}$ represents the input feature. The output feature after three convolutional layers is ${\text{Conv}}_{3}\left({\text{Conv}}_{2}\left({\text{Conv}}_{1}\left(\hat{x}\right)\right)\right)$. After the sum operation with $WF\left(\hat{x}\right)$, the final output feature $DSF\left(\hat{x}\right)$ is obtained.

## 4 Experiments

### 4.1 Experimental setup

#### 4.1.1 Dataset

We constructed a new dataset called the Mouse Cardiac Infarction Image Dataset, which includes 243 images. According to the mouse of myocardial injury, we collected 243 cardiac slice images from C57BL/6 mice. These images were captured by the camera with a size of 6,000 × 4,000 pixels. After coronary artery ligation surgery, their cardiac were removed and sliced. Each mouse's cardiac was cut into five slices. Different slices from the same mouse cardiac are placed on the same glass slide, and the camera captures them as one image. To quantitatively measure the infarct size, we performed triphenyl-tetrazolium chloride (TTC) and Evans blue staining on each cardiac slice. TTC staining colors active cardiac tissue red to depict risk and infarct areas (24). Meanwhile, Evans Blue will dye the surviving cardiac tissue blue. Therefore, non-ischemic areas are colored blue, dangerous areas are colored red, and infarcted areas are marked white (2). We have implemented a series of preventive measures to ensure the high quality of annotations in image processing (25). The annotator received training from a senior expert in mouse cardiac image segmentation. We divided the images into balanced batches covering different experimental time points and annotated them according to the batches. This batch-based strategy aims to effectively reduce internal bias in the dataset that may be introduced due to the experience growth of annotators during the annotation process. In addition, this dataset is the first mouse cardiac image dataset that covers the left ventricle, danger zone, and infarct zone.

#### 4.1.2 Implementation details

All networks used in our experimental setup were implemented using pytorch on servers with Tesla V100-PCIE-32GB. The experiment is performed with a train-val-test split of 70–15–15 at the mouse level. The input patch size received by the network is 256 × 256. And set the initial learning rate to 1e-3. We trained a model with 60 epochs, batch size of 2, using the AdamW optimizer, momentum = 0.9 and weight decay rate = 0.05.

To illustrate the effectiveness of the proposed approach in cardiac segmentation, the DICE similarity coefficient, Hausdorff distance (HD) and Interquartile Range (IQR) are used as performance measures.

### 4.2 Quantitative evaluation

Table 1 shows the advantages of our method. The results indicate that our proposed DUCNet achieved good segmentation results in the mouse cardiac data set. As shown in Table 1, our method outperforms other algorithms in Dice score, reaching an average Dice of 80.68%. This score is significantly higher than DSCNet's 78.98%, indicating that our proposed method performs better compared to the method based on tubular feature design. It should be noted that our method achieves a Dice value of 80.13% for IS segmentation, which is 2.43% higher than DSCNet, further verifying the superiority of our model on irregular U-shaped structures. In addition, our method performs well compared to transformer-based algorithms. Our average Dice is 8.27% higher than TransUNet, indicating that our model is more effective at capturing key features when processing mouse cardiac data sets. Further analysis shows that there is a 0.48 reduction in HD value with our model compared to DSCNet, mainly due to the U-shaped convolution we designed for the U-shaped structure of the data. Compared to the serpentine convolution of DSCNet, our design is more adaptable to the features of U-shaped structures in the data, effectively compensating for potential information loss issues.

**Table 1:** Comparison with other segmentation networks-all mask val test.

Network	Volumetric (%) ↑				Distance ↓ Avg_HD
	Avg_dice	Lv_dice	Arr_dice	Is_dice	
MultiResUNet (29)	39.55	30.22	47.93	40.51	31.82
LeViT-UNet192 (30)	68.95	79.66	66.46	60.73	14.99
MISSFormer (31)	69.12	79.88	67.42	60.06	14.79
Unet (14)	70.33	80.08	67.92	63.00	17.58
SwinUnet (32)	71.62	80.26	69.75	64.87	14.41
Transunet (33)	72.41	80.68	69.33	67.21	14.26
DSCAU-Net (34)	73.15	81.41	70.82	67.23	13.97
VM-unet (21)	73.89	80.51	70.33	70.85	16.02
EEMSNet (22)	74.22	69.46	**81.10**	72.11	20.28
ResUNet (35)	74.76	82.27	71.24	70.78	13.61
UCTransNet (36)	76.70	82.49	73.57	74.05	13.12
Unet++ (18)	77.09	82.80	74.33	74.15	13.04
Attention-UNet (16)	77.80	83.11	74.85	75.45	**12.87**
DSCNet (11)	78.98	83.64	75.61	77.70	14.23
DUCNet (ours)	**80.68**	**84.22**	77.69	**80.13**	13.75

### 4.3 Qualitative evaluation

**Figure 4:**
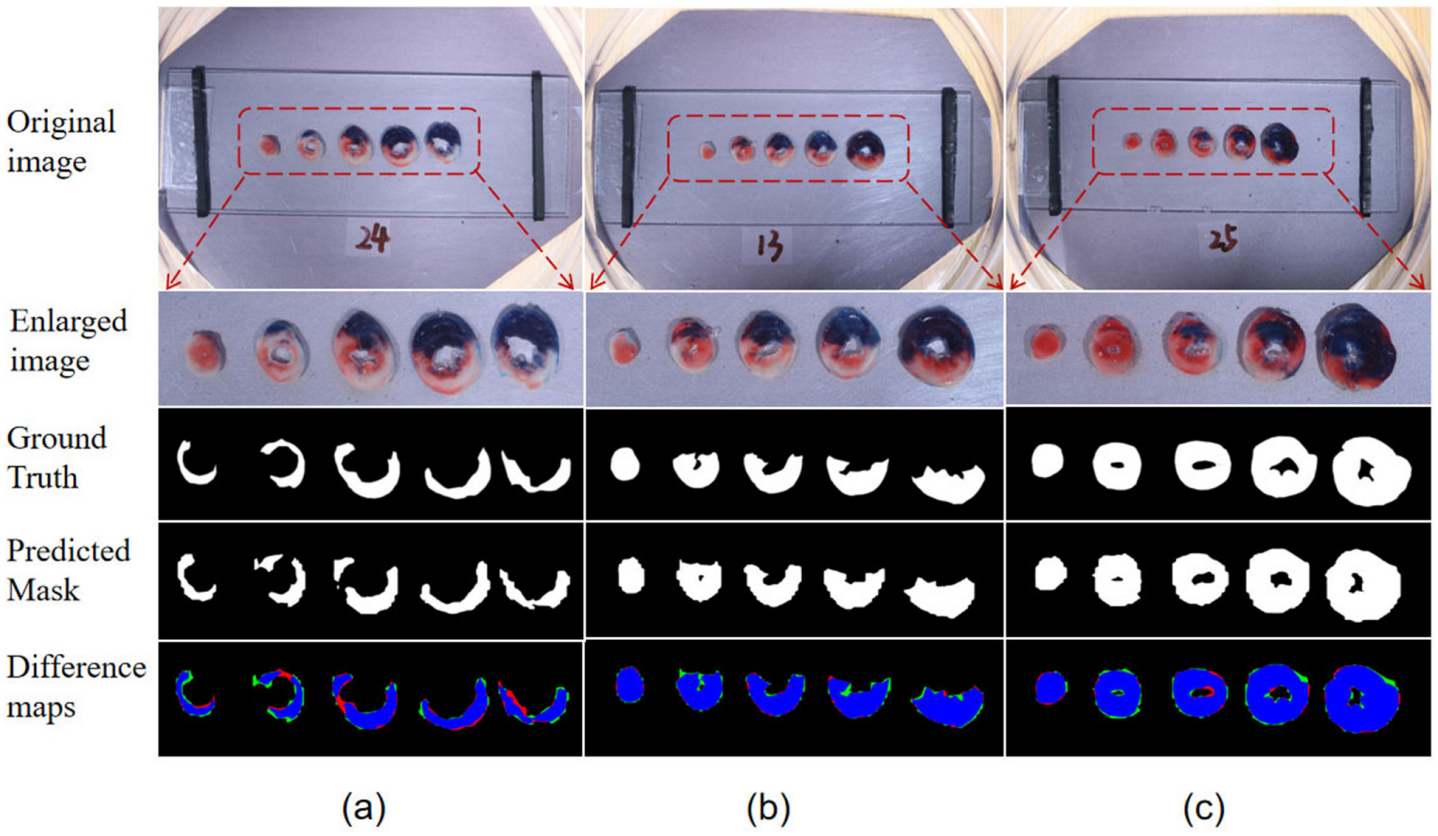
Segmentation examples of the proposed method. **(a)** Segmentation of infarct area. **(b)** Segmentation of area at risk. **(c)** Segmentation of Left ventricular. In the difference maps: blue (true positive), green (false positive), red (false negative). To provide a clearer view of our segmentation results, we have enlarged the segmentation result images.

This article proposes a DUCNet for the segmentation of mouse cardiac slice images. The performance of the proposed DUCNet in the mouse cardiac slice image segmentation task is shown in Figure 4. Specifically, our model performs well in IS segmentation, as shown in Figure 4a. This is due to our proposed dynamic U-shaped convolution mechanism, which can effectively capture and process irregular U-shaped regions. This convolutional structure enables the model to locate and segment the IS more accurately, improving the accuracy and precision of the segmentation. At the same time, our model also performed well in the ARR and LV segmentation tasks, as shown in the results in Figures 4b, c, where false positive regions (green) and false negative regions (red) were significantly reduced. This indicates that our model can effectively reduce the error detection rate while maintaining high accuracy, improving its reliability and stability in ARR and LV segmentation tasks.

**Figure 5:**
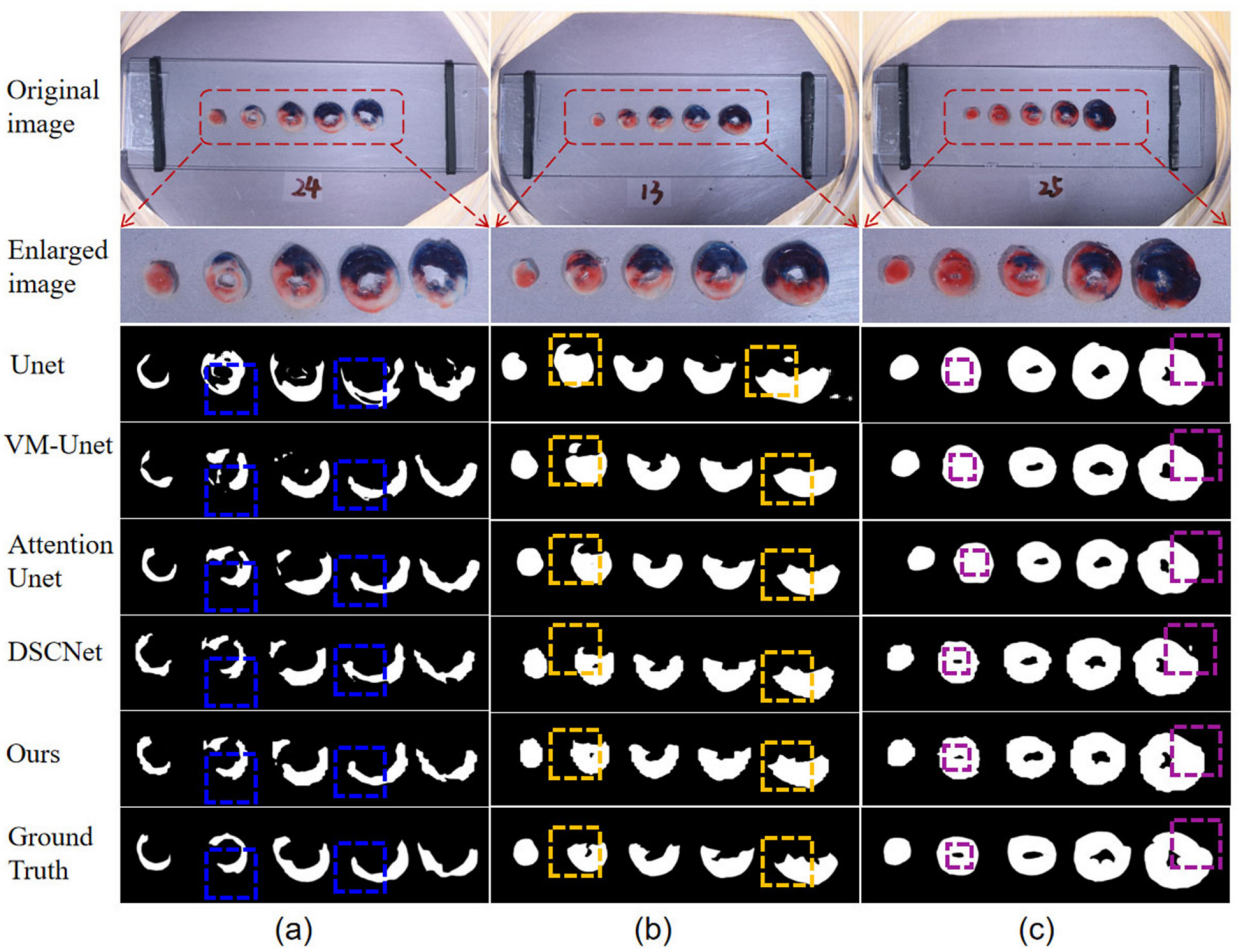
Prediction area of different networks. **(a)** Segmentation of infarct area. **(b)** Segmentation of area at risk. **(c)** Segmentation of left ventricular. The blue box represents the comparison area for the IS segmentation, the orange box represents the comparison area for the AAR segmentation, and the purple box represents the comparison area for the LV segmentation. In order to provide a clearer view of the segmentation results, we enlarged the segmentation result image.

To demonstrate the performance of different models in mouse cardiac slice image segmentation tasks, we compared U-Net, VM-Unet, Att-Unet, DSCNet, and DUCNet (the model proposed in this paper). Figure 5 shows an example of segmentation of three mouse cardiac slice images. In the segmentation of the IS (Figure 5a), the U-Net and VM-Unet models showed difficulty in segmentation, resulting in a significant number of false positive areas. In contrast, our model, by introducing U-shaped dynamic convolution, is able to identify irregular U-shaped infarction area features better and exhibits better performance. For the segmentation of the AAR, as shown in Figure 5b, our DUCNet successfully segmented the risk zones within the orange box. This is mainly due to the adoption of the WF branch, which improves the model's recognition and resolution of object boundaries and details.

The segmentation results of U-Net, VM-Unet, Attention-Unet, and DSCNet showed that they misclassified other areas as risk zones. Furthermore, according to the results in Figure 5c, although all models were able to successfully segment the LV, in the second slice, the U-Net, VM-Unet, and Attention-Unet models were unable to accurately determine the left ventricle. DSCNet failed to accurately identify edge information in the segmentation of the fifth slice, resulting in misjudging other regions as the LV. Specifically, our proposed DUCNet method performs well in processing complex mouse cardiac slice images, providing clear and accurate segmentation results. This is because DUCNet utilizes dynamic U-shaped convolution to capture detailed irregular U-shaped features better, and the designed WF branch extends the model's receptive field, enabling it to identify and segment target objects more accurately.

In the analysis of mouse cardiac tissue slice images, in addition to achieving automatic segmentation of the myocardial infarction area, our segmentation model can further be applied to the automatic calculation of the myocardial infarction area. Accurate calculation of the infarction area is of great clinical significance for assessing the severity of myocardial injury, the extent of infarction, and the effectiveness of treatment (26). Traditionally, the calculation of the infarction area often relies on manual measurements by professionals using ImageJ (11), which is not only time-consuming but also prone to subjective bias. However, the automated approach we propose can significantly improve both the efficiency and accuracy of the calculation.

**Figure 6:**
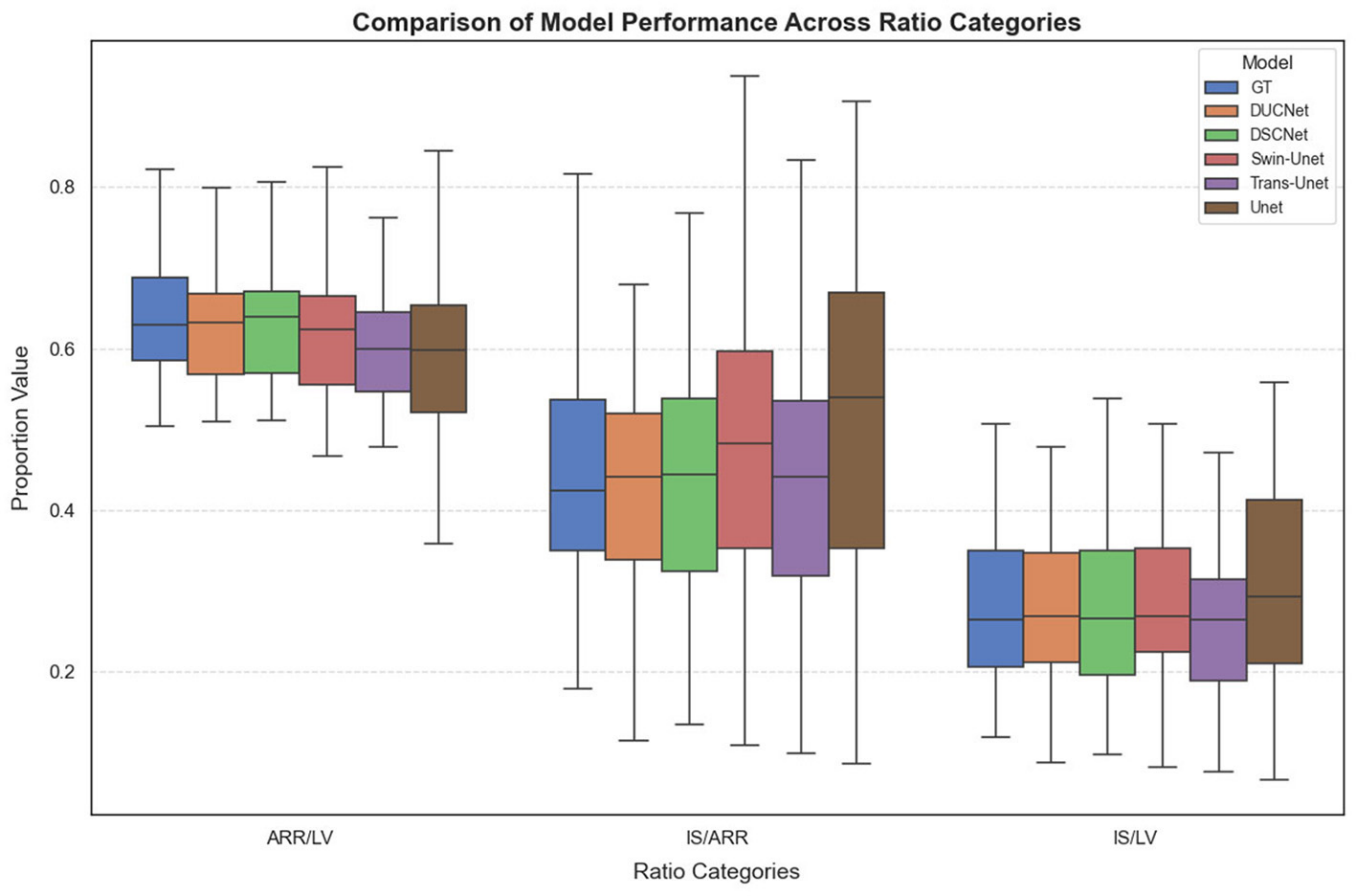
The proportional values of ARR/LV, IS/ARR, and IS/LV.

To present the performance of different models in terms of stability and application value, we analyze the results of various models for predicting ARR/LV, IS/ARR, and IS/LV from the median and IQR. ARR/LV and IS/ARR are valuable parameters in animal medicine research (27). The ARR/LV ratio is used to evaluate the extent of left ventricular damage after myocardial infarction, in order to quantify the degree of impact of myocardial infarction on the left ventricle (28). The IS/ARR ratio is used to measure the proportion of infarct area (IS) in the risk area (ARR). The higher the ratio, the larger the infarct area and the more severe the condition may be. In addition, IS/ARR can also reflect the therapeutic effect, for example, after effective intervention, a decrease in this ratio may mean a reduction in the infarct area and improvement in the condition. As shown in Figure 6, we first analyze from the median perspective. In the ARR/LV category, DUCNet and SwinUnet exhibit median values that are closer to the GT median line compared to other models, particularly DSCNet, TransUnet, and Unet. However, in the IS/ARR category, DUCNet shows a median that is closer to the GT median than SwinUnet. Additionally, DUCNet also performs excellently in the IS/LV category, with a median very close to the GT median. Therefore, from the median perspective, DUCNet demonstrates more stable performance across different categories and its predictions are closer to the real values compared to other networks.

### 4.4 Ablation analysis

The purpose of ablation research is to analyze the impact of various factors on model performance. Specifically, we explored the roles of WF branch, AG module and Res branch in model performance. In addition, we also analyzed the impact of the internal design of the WF branch on model performance.

In Table 2, we presented the ablation study results of the proposed DUCNet model under different module configurations. Specifically, we evaluated the impact of the WF branch, AG module, and Res branch on the model's performance. When only the WF branch was used, the model's average Dice value was 80.36%, which was 2.07% higher than when only the AG module and Res branch were used. Notably, the Dice value of the IS region increased significantly by 3.14%, indicating that the WF branch effectively recognized the features of irregular U-shaped structures and played a key role in improving the model's performance. When the WF branch, AG module, and Res branch were used together, the average Dice value reached 80.68%, which was 0.32% higher than when only the WF branch was used (80.36%). This result suggested that the combination of the AG module and Res branch had a positive impact on the model's segmentation performance. In particular, in the ARR region, the Dice value improved from 76.94 to 77.69%, further indicating that the combined modules significantly improved feature extraction and segmentation accuracy in these complex regions. Additionally, we observed that when the WF and AG modules were used together, the introduction of the Res branch increased the model's average Dice value from 79.38 to 80.68%. This indicated that the Res branch, by introducing residual connections, effectively alleviated the vanishing gradient problem, allowing the network to learn deeper feature representations, thereby further enhancing segmentation accuracy. When the WF and Res branches were used together, the introduction of the AG module increased the model's average Dice value from 79.63 to 80.68%. This indicated that the AG module, by suppressing irrelevant regions in the mouse cardiac slice images, highlighted the significant features of specific local regions, further improving the segmentation performance.

**Table 2:** Ablation study on the impact of the different module configurations.

WF	AG	Res	*Avg* (*%*)	*LV* (*%*)	*ARR* (*%*)	*IS* (*%*)
✓			80.36	84.08	76.94	80.07
✓	✓		79.38	83.91	75.51	78.72
✓		✓	79.63	84.03	76.20	78.65
	✓	✓	78.29	83.28	74.67	76.93
✓	✓	✓	**80.68**	**84.22**	**77.69**	**80.13**

## 5 Conclusion

In this study, we propose the DUCNet model designed explicitly for the segmentation and quantification of mouse cardiac slices. We propose a dynamic U-shaped convolution method that effectively captures the features of irregular U-shaped structures while highlighting local key features and suppressing irrelevant information through the AG suppression mechanism in skip connections. In addition, we constructed a detailed annotated dataset of mouse cardiac slices, including 243 images and a total of 1,215 slices, providing a solid foundation for this study. We conducted comprehensive experimental validation on the dataset, and the results showed that our proposed method has an average Dice coefficient of 80.63%, which is superior to current leading models such as VM-Unet, DSCNet, and TransUnet. Although the proposed DUCNet algorithm outperforms existing algorithms in terms of results, its model efficiency has limitations. The proposed algorithm has 239.04M parameters and 263.13G FLOPs, compared to U-Net's 28.99M parameters and 50.74G FLOPs. Therefore, future work will focus on model lightweighting.

## Data Availability

The original contributions presented in the study are included in the article/supplementary material, further inquiries can be directed to the corresponding authors.
